# Resistance to African swine fever virus among African domestic pigs appears to be associated with a distinct polymorphic signature in the *RelA* gene and upregulation of *RelA* transcription

**DOI:** 10.1186/s12985-024-02351-9

**Published:** 2024-04-24

**Authors:** Patrick N. Bisimwa, Juliette R. Ongus, Ronald Tonui, Espoir B. Bisimwa, Lucilla Steinaa

**Affiliations:** 1 Molecular Biology Laboratory, Department of Animal Sciences and Production, Université Evangélique en Afrique, Bukavu, Democratic Republic of Congo; 2 Institut Supérieur de Dévelopement Rural de Kaziba, Kaziba, Democratic Republic of Congo; 3 Department of Medical Laboratory Sciences, Jomo Kenyatta University of Agriculture and Technology, Nairobi, Kenya; 4 Biotechnology Laboratory, Departement of Molecular Biology and Biotechnology, Pan African University Institute of Basic Sciences, Technology and Innovation, Nairobi, Kenya; 5 Animal and Human Health Program, International Livestock Research Institute, Nairobi, Kenya

**Keywords:** African swine fever virus, Infection, Pigs, Polymorphisms, RelA, Disease resistance

## Abstract

African swine fever virus (ASFV) is a highly contagious and fatal hemorrhagic disease of domestic pigs, which poses a major threat to the swine industry worldwide. Studies have shown that indigenous African pigs tolerate ASFV infection better than European pigs. The porcine v-rel avian reticuloendotheliosis viral oncogene homolog A (RelA) encoding a p65 kD protein, a major subunit of the NF-kB transcription factor, plays important roles in controlling both innate and adaptive immunity during infection with ASFV. In the present study, RelA genes from ASFV-surviving and symptomatic pigs were sequenced and found to contain polymorphisms revealing two discrete RelA amino acid sequences. One was found in the surviving pigs, and the other in symptomatic pigs. In total, 16 nonsynonymous SNPs (nsSNPs) resulting in codon changes were identified using bioinformatics software (SIFT and Polyphen v2) and web-based tools (MutPre and PredictSNP). Seven nsSNPs (P374-S, T448-S, P462-R, V464-P, Q478-H, L495-E, and P499-Q) were predicted to alter RelA protein function and stability, while 5 of these (P374-S, T448-S, P462-R, L495-E, and Q499-P) were predicted as disease-related SNPs.

Additionally, the inflammatory cytokine levels of IFN-α, IL-10, and TNF-α at both the protein and the mRNA transcript levels were measured using ELISA and Real-Time PCR, respectively. The resulting data was used in correlation analysis to assess the association between cytokine levels and the RelA gene expression. Higher levels of IFN-α and detectable levels of IL-10 protein and RelA mRNA were observed in surviving pigs compared to healthy (non-infected). A positive correlation of IFN-α cytokine levels with RelA mRNA expression was also obtained. In conclusion, 7 polymorphic events in the coding region of the RelA gene may contribute to the tolerance of ASFV in pigs.

**Supplementary Information:**

The online version contains supplementary material available at 10.1186/s12985-024-02351-9.

## Introduction

African swine fever (ASF) is a hemorrhagic and fatal infectious disease that causes rapid death in domestic pigs (*Sus scrofa*), originally described for the first time in Kenya [1]. It has lately spread across the globe, causing huge economic losses. ASF is caused by the African swine fever virus (ASFV), a large enveloped and structurally complex double-stranded DNA virus. The virus has a linear genome with sizes between 170 and 193 kbp, which encodes 150–167 open reading frames dependent on the strain [2]. ASF is a major constraint for pig farming in Sub-Saharan Africa [3].

Previous studies were conducted in different parts of DRC, some have identified the circulation of genotypes I, IX and XIV the causative agent of ASF outbreaks in in the Western, Central and Northern parts [4]. Other recent studies conducted in the South Kivu province east of DRC showed genotypes IX circulating in asymptomatic pigs while genotype X was found in symptomatic domestic pigs in outbreaks in the region [5, 6].

African domestic pigs (African indigenous pigs) are more susceptible to ASF, with mortality rates of up to 100% in infected herds compared to wild pigs such as warthogs and bushpigs [7, 8]. Ticks of the species *Ornithodoros moubata* and *Ornithodoros erraticus* can transmit the virus [9]. The difference in resistance to ASFV between domestic and wild pigs is attributed to genetic variation in the hosts and has been suggested to be due to differences in the ability of the virus to modulate the immune responses in these different hosts [10].

Previous studies have shown evidence that African indigenous domestic pigs in most African countries are less susceptible to ASFV infection than European pigs [11, 12]. The wild pigs and the African domestic pigs have been exposed to ASFV for many more generations than the European pigs and have had the time to adjust to the surrounding pathogens simply by genetic evolution, which may explain the resistance to ASFV

African swine fever virus primarily infects macrophages, which are major targets for in vivo viral replication [13]. When infected and activated by ASFV, macrophages drive the immune response by secreting a wide range of mediators, including pro-inflammatory cytokines (e.g., IFN type I and TNF-α) as well as those that facilitate the development of specific immune mechanisms (IL-10, IL-12) through activation of both the Th1 and Th2 responses [14]. An in vitro study comparing growth curves of ASFV in macrophages from wild pigs and domestic pigs showed a similar capacity of the macrophages in supporting ASFV growth [7], indicating that other mechanisms may explain differences in virus susceptibility than differences in infection and propagation. Various studies have demonstrated an association between ASFV pathology and overexpression of cytokines such as IFN alpha (IFN-α) and tumor necrosis factor-alpha (TNF-α) in domestic pigs [15, 16]. Another study has reported that ASFV virulent such as Armenia/07 blocks the synthesis of IFN-β, a key mediator between the innate and adaptive immune response [17]. Additionally, the nuclear factor kappa-B (NF-κB) transcription factor plays an important role by controlling the transcription of these cytokines (IFN-α and TNF-α) [18]. The mammalian NF-κB transcription factor comprises RelA (p65), RelB, p50/NF-κB1, p52/NF- κB2, and c-Rel (Supplementary Fig. 1). The heterodimer of RelA (p65) and p50 is the most commonly found heterodimer complex among NF-κB and is the functional component participating in nuclear translocation and activation of NF-κB [19].

Like other NF-κB sub-units, the *RelA* gene product is composed of an N-terminal REL-homology domain (RHD) responsible for DNA binding, dimerization, and NF-κB/REL inhibitor interaction, and a C-terminal transactivation domain (TAD) (Supplementary Fig. 1), which interacts with the basal transcription complex, involving several coactivators of transcription, such as TATA-binding protein (TBP), Transcription Factor IID (TFIIB) and the cAMP-response element binding protein (CREB-CBP) [20]. The NF-κB gene encodes one of the subunits of the NF-κB heterodimer, which is involved in a variety of intracellular pathways, among others; it invokes overreaction of the host immune system with devastating effects [19]. Polymorphic variants found in the warthog *RelA* gene have been suggested as a potential genetic factor to explain the surviving phenotype upon infection with ASFV [10], but this was never firmly established. In line with this, variation in the porcine *RelA* gene may explain why some domestic pigs are more resilient to ASFV infection than others. Furthermore, several proteins expressed by ASFV have been experimentally validated for their ability to suppress the immune system in vitro by reducing the interferon response and the NF-κB activation [2, 20]. This creates a favorable environment for viral replication [21].

The availability of data on immunogenetic features underlying the immune responses in domestic pigs is scarce, and most studies are limited to analyses in domestic pig cells. Among the ASFV genes, A238L substitutes for NF- κB Inhibition Activator (NF-κBIA) by binding to the p65 (*RelA*) sub-unit of NF-κB preventing the ability of NF-κB to be activated [22, 23]. Additionally, the viral protein A238L was shown to act as inhibitor for the activation of the transcriptional co-activator p300/cbp1 [18, 24]. Similarly, previous studies showed that A238L gene binds and inhibits calcineurin phosphatase and activation of NFAT transcription factor [25, 26]. Some other recent papers have investigated other ASFV inhibitors of NF-kB including pD345L and I10L by targeting IKK kinase activity [27, 28]. In addition, the ASFV inhibitor of apoptosis (IAP) has been shown to activate the transcription factor NF-κB [29].

The present study aimed to identify the polymorphisms in the *RelA* gene in ASFV-surviving (non-symptomatic but infected pigs) versus susceptible (symptomatic and infected) domestic pigs from the field and to assess the correlation of *RelA* mRNA expression with cytokine levels. It is envisaged that the findings of this study will promote a better understanding of genetic factors underlying ASF susceptibility.

## Materials and methods

### Study area

**Fig. 1:**
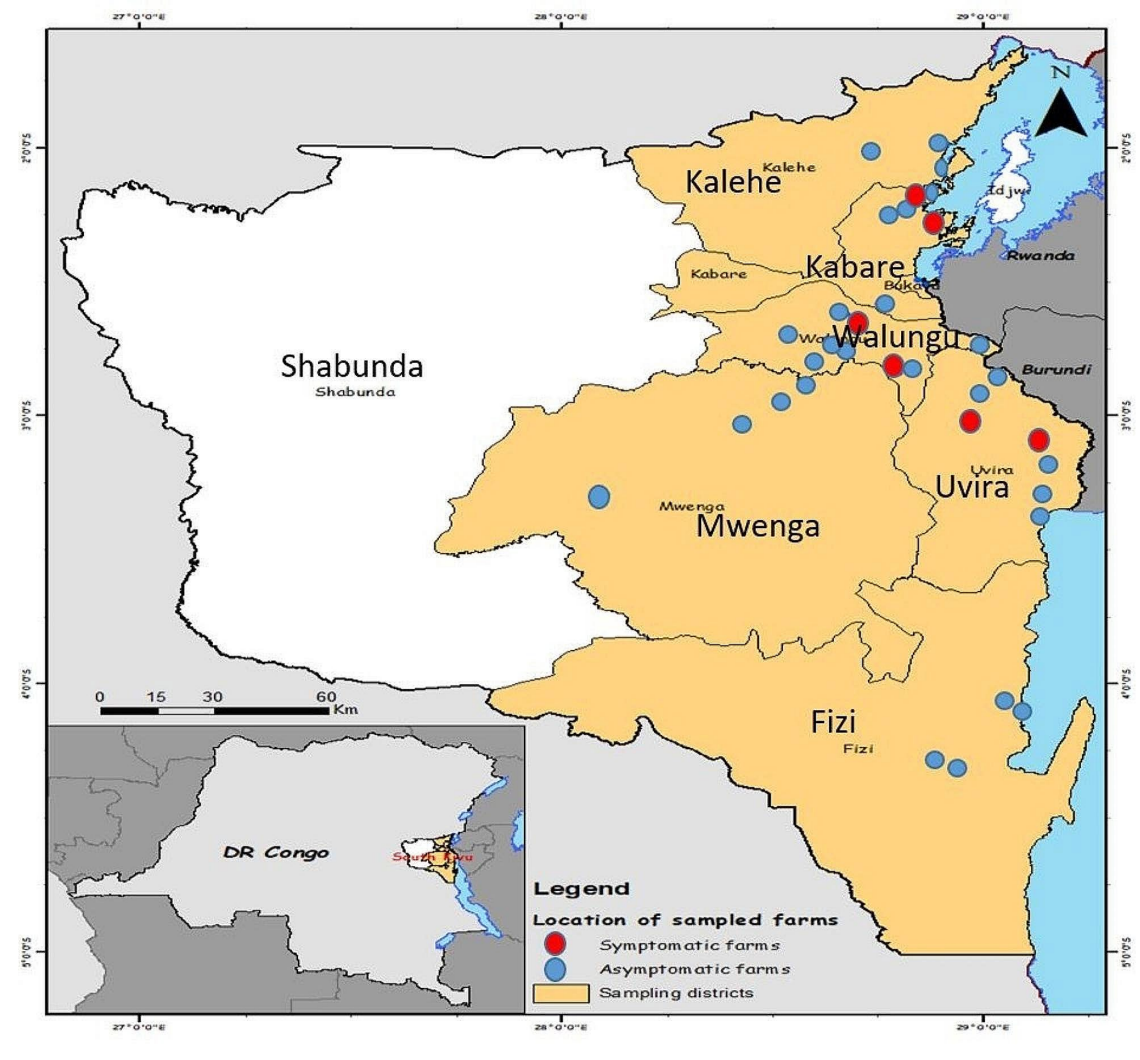
Map of the South Kivu province showing the study location and collection points of samples (Drawn with Arc-GIS)

The study was conducted in six different districts in the South Kivu province located in the Eastern part of the Democratic Republic of Congo, including Fizi, Kabare, Kalehe, Mwenga, Uvira, and Walungu. The collection points in each district are shown in Fig. 1. This vast region has an area of 66.814 Km^2^, located between longitudes 26° 10’ 30” and 29° 58’ East and latitudes 00’ 58” North and 4° 51’ 21” South.

As part of a larger survey to elucidate the presence of ASFV in apparently healthy [5], six districts (Kabare, Kalehe, Fizi, Mwenga, Uvira, Walungu) in DRC Congo were revisited after reports that ASFV-infected pigs had recovered from the disease, to assess the relationship of ASFV tolerance with *RelA* polymorphisms and cytokine levels.

### The origin, PCR, and ELISA status of the tested samples

In total, 90 samples from the districts mentioned above were subjected to PCR analysis and ELISA tests to assess if the pigs had been or were infected with ASFV along with a clinical assessment, such as high fever, severe recumbency, cyanotic skin on ears, bleeding from bodily orifices, and difficulty of breathing [5]. Any PCR positive sample was considered as infected pigs while an ELISA positive test was considered as previous infection with non-active infection. In addition, samples were selected based on geographical location, and the criteria were that they should be ELISA or PCR positive to proceed in the study. From this number, 60 of the PCR-positive samples (with the highest DNA yield) were amplified for the *RelA* gene, of which 40 samples resulted in *RelA* sequences of sufficient quality.

### Collection of samples

Whole blood (4 ml) was collected in EDTA tubes from pigs (all local breed) with clinical symptoms of ASF collected in December 2018 (high fever, severe recumbency, cyanotic skin on ears, bleeding from bodily orifices and difficulty of breathing) and from apparently healthy pigs collected in June 2017. Samples were stored at -20 °C.

### Genomic DNA isolation

Porcine gDNA was extracted directly from 200 μl of whole blood using a DNeasy® Blood & Tissue kit (Qiagen, Hilden, Germany) according to manufacturer’s instructions. The gDNA concentrations were determined by spectrophotometry using Nanodrop (PCR^max^ Lamda, UK). The integrity of the DNA was measured on a 1% agarose gel stained with SYBR® Green I Nucleic Acid Gel Stain (10,000x, Invitrogen). The infection status of the animals was determined by polymerase chain reaction (PCR) and indirect enzyme-linked immunosorbent assay (ELISA), respectively, as reported in our previous work [5].

### Primer design and PCR amplification of the porcine RelA gene

The genomic sequence of the *RelA* gene was retrieved from the GenBank database’s *Sus scrofa* isolate TJ Tabasco Breed Duroc chromosome 2 Sscrofa 11.1 (NC_010444.4). Primer-Blast tool was used to design 6 pairs of primers to amplify complete and overlapping exons and adjacent introns of the *RelA* gene (Table 1) while the expression level of RelA gene was assessed by primers described in Table 2. Primers were synthesized by Macrogen Europe (Amsterdam, Netherlands).

**Fig. 2:**
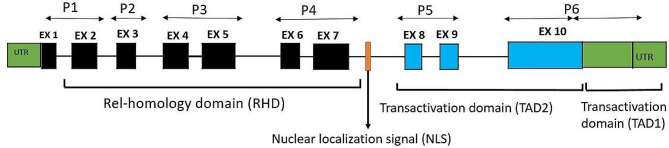
The genomic structure of the porcine RelA gene. Rectangles represent exons and are drawn on a scale. Black rectangles represent exons that compose the Rel-homology domain, while blue rectangles represent exons of the transactivation domain. Green rectangles show the untranslated regions found in the RelA cDNA. P1 represents the primers location for the sequenced exons to P6. Ex: exons, P: primer

The porcine *RelA* gene is located on chromosome 2 between positions 6.594.869 to 6.602.684 with 7816 base pair in length. The genomic sequence includes 10 exons and 9 introns, and additional 5 ’and 3’ untranslated regions (Fig. 2). It comprises 1,662 nucleotides encoding a protein of 554 amino acids in length. In this study, five overlapping primer sets covering all 10 exons were designed to amplify and fully sequence all 10 exons of the *RelA* gene to identify potential polymorphisms in pigs. Primers were placed in conserved areas, facilitating amplification of any polymorphisms in the 10 exons. It was assumed that nonsynonymous single nucleotide polymorphisms (nsSNP) within the coding sequence (exons) would be of significant value as they could affect protein function.

For amplification of the target sequence, 1 μl of gDNA (50 ng/μl) was mixed with 10 pmol of each primer into 12.5 μl of PCR master mix (2xAccuPower Tap PCR Master Mix, Bioneer) containing 1U of TaqDNA polymerase (Bioneer, USA), 250 μM of dNTPs each, 1x reaction buffer with 1.5 mM MgCl_2_ and trace of tracking dye. The final reaction volume was brought to 25 μl with nuclease-free water.

The PCR cycling conditions used comprised an initial denaturation step of 5 min at 95 °C followed by 35 cycles comprising denaturation at 95 °C for 30 s, primer annealing at 55 °C for 15 s, and extension at 72 °C for 1 min. The final extension was done at 72 °C for 5 min. The PCR programs for all the primers were optimized and were identical for each target sequence. PCR products were resolved on a 1.2% agarose gel stained with SYBR® Green I Nucleic Acid Gel Stain (10,000x, Invitrogen), run in an electrophoresis chamber at 60 volts/cm for 40 min, and visualized using UV light. Amplicons of the expected size were purified using QIAquick PCR purification Kit (Qiagen, Germany) following the manufacturer’s recommendation and were sent for sequencing (Macrogen Europe Inc., Amsterdam, Netherlands).

**Table 1:** Primers for characterization of the porcine RelA gene

Primer name	Primer Sequences 3’ to 5’	Annealing temperature(°C)	Purpose	Product length
P1	F-TCAGACCTCTTCCCCCTCATCT R-GAGCTCACCTGTGGATGCAG	58	Polymorphism	600bp
P2	F-AAAGAGGGGTTGTGAGCTTG R-CACCTCGATGTCCTCTGATG	55	Polymorphism	464bp
P3	F-GGTGGCAGGGAACTGATTTA R-GACACAGATTTGCGTCCACT	55	Polymorphism	826bp
P4	F-GCTGTTGTCCTCATGAACCT R-TGAGATTATGTCGCTTCGCC	55	Polymorphism	764bp
P5	F-TCCAGCTGGGAAGAGCAA R-AGGAAGTCACCGGTTCCT	55	Polymorphism	600bp
P6	F-TCTCCTCCAGCTCCCCA R-AGAAGGGCTGAGAAGTCCAT	51	Polymorphism	620bp

**Table 2:** Primers for quantification of mRNA expression

Primer name	Primer Sequences 3’ to 5’	Annealing temperature(°C)	Purpose	Product length
18SrRNA	F-GACGTGACTGCTCGGTGC R-AACTCGACCGAGGGCACAAG	62	Housekeeping	144bp
RelA qPCR	F-AGTACCCTGAGGCTATAACTCGC R-TCCGCAATGGAGGAGAAGTC	62	qPCR	133bp

### Polymorphisms and prediction of their effect on protein function

The amplicon sequences were trimmed, and quality checks were done using CLC main Work Bench version 7.8.1 software (https://digitalinsights.qiagen.com). The nucleotide sequences and the translated amino acid sequences from surviving pigs were aligned and compared with sequences from symptomatic (sick) pigs by using the ClustalW (http://www.ebi.ac.uk/Tools/msa/clustalo/) tool in the MEGA 7 software. Potential polymorphism sites were detected by sequence comparisons using the DNAstar software (DNAstar Inc., Madison, WI, USA) and the DNA Sequence Polymorphism (DnaSP) version 6.12.03 (Universita de Barcelona).

*In silico* analyses were performed to predict the functional effects of amino acid changes using the software: Sorting Intolerant From Tolerant (SIFT) [30] and Polymorphism Phenotyping V2 (Polyphen v2) [31]. SIFT is a sequence homology-based prediction tool used to identify potential amino-acid substitutions that may affect biological functions through protein structural modifications. From SIFT analyses, scores between 0.00 and 0.05 were considered damaging, while scores beyond the threshold fixed at 0.05 were predicted to be neutral. PolyPhen-2, a physical and evolutionary comparative tool, was used to predict the effect of amino acid substitutions on protein structure and function. The scores were classified as probably damaging (≥ 0.85), possibly damaging (0.5–0.84), and benign (< 0.5). In this study, we assumed that any SNP with scores “probably damaging” and “possibly damaging” would affect protein functions.

### Prediction of disease-related amino acid substitutions and their effect on protein stability

The web-based tools, MutPred (http://mutpred.mutdb.org/) and PredictSNP http://loschmidt.chemi.muni.cz/predictsnp are online server tools which integrate genetic and molecular data to predict the detrimental effect of amino acid substitutions in a mutant protein [32, 33]. The outputs from these two tools were combined to improve the prediction accuracy. For MutPred, scores with g-value (probability for pathogenic amino acid substitutions) > 0.50 and *p*-value < 0.05 were considered actionable hypotheses (a given amino acid change with pathogenic effect). In contrast, the scores with g-value > 0.70 and *p*-value < 0.05 are referred to as confident hypotheses (with no pathogenic effect). However, for Predict SNP, scores with *p*-values (probability for deleterious of given amino acid substitution) <-1 to 0: neutral; *p*-value: 0 to + 1: deleterious.

In addition, MUPro [34] and I-Mutant 3.0 (http://gpcr2.biocomp.unibo.it/cgi/predictors/ are support vector machine-based tools that were used for predicting the effect of nonsynonymous amino acids substitutions on protein stability. MUPro predicts the energy change value and yields a confidence score between − 1 and 1 to be used for calculating the confidence of the prediction. Scores < 0 suggest that the amino acid change decreases protein stability, whereas scores > 0 indicate increased protein stability [35]. Moreover, the outputs of the I-Mutant prediction method of protein stability changes are based on the value of free energy change: largely destabilizing ( < − 0.5 Kcal mol − 1), largely stabilizing (> 0.5 Kcal mol − 1), or weakly stabilizing or destabilizing (− 0.5 ≤ Delta Delta Energy (DDG) ≤ 0.5 Kcal mol − 1).

### Relative quantification of IFNα, IL10, and TNF-α gene expression by real-time PC

RNA was extracted from blood samples using PureLink™ RNA Mini Kit (Thermo Fisher, Ambion Life Technology, California) following the manufacturer’s recommendations. The RNA content and purity were estimated using the Nanodrop (PCRmax Lambda) spectrophotometer and the 260/280 nm ratio, respectively. cDNA was done using RevertAid First Strand cDNA Synthesis Kit (Fermentas, Thermo Scientific, #1622) following the manufacture’s protocol. Briefly, 10 μg of the total RNA and 1 μl of oligo (dT) primer were added to 8 μl of RevertAid master mix, and the volume was brought up to 20 μl. The reactions were incubated for 5 min at 25 °C followed by 60 min at 42 °C. The reaction was heated at 70 °C for 5 min in a thermocycler machine (ProFlex, PCR system, Applied Biosystem). The obtained cDNA samples were stored at − 20 °C until further use. For quantitative PCR analysis, expressions of all the genes (IL-10, IFN-α, and TNF-α) were quantified using specific primers, as presented in Table 3. Values were normalized to 18 S rRNA, a housekeeping gene, which is a common choice as a reference gene (Table 2).

**Table 3:** Primer sequences used in this study to quantify the mRNA expression of the selected cytokines

Cytokines	Oligonucleotide sequences (5’- 3’)	Ref sequence Acc. number	Reference
IFN-α	FCCAGGTCCAGAAGGCTCAAG	NM_214393	[36]
	R-GCAGCCGAGCCCTCTGT		
IL-10	GCATCCACTTCCCAACCA	NM_214041	[37]
	CTTCCTCATCTTCATCGTCAT		
TNF-α	TGGCCCCTTGAGCATCA	NM_214022	[37]
	ACGGGCTTATCTGAGGTTTGAG		

The specificity of the qPCR was assessed by the melting curves generated after amplification. All the samples were run in triplicates. The relative expression of each sample was calculated using the method suggested by Livak and Schmittger [38]. The qPCR mix comprised 10 μl of 2X Luna Universal qPCR Master Mix (New England, BioLabs Inc.), 2 μl of cDNA, and 0.5 μl containing 10 pmol of each forward and reverse primer were added; the volume was topped up to 25 μl with nuclease-free water. Amplification of ASFV was accomplished in a LightCycler ® 96 (LifeScience, Roche) with the following conditions: 50 °C for 2 min, one cycle (uracil N-deglycosylase digest); 95 °C for 1 min, 95 °C for 15 s, 62 °C for 60 s, 40 cycles. For specificity of the PCR, information on melting curves was collected continuously from 65 °C to 95 °C.

### Quantification of IL-10, IFN-α, and TNF-α levels in serum samples by enzyme-linked immunosorbent assay (ELISA)

The level of IFN-α, TNF-α, and IL-10 cytokines in symptomatic, surviving, and healthy pigs was evaluated by quantitative sandwich-type enzyme-linked immunosorbent assay (ELISA) kits (Eagle Biosciences, Inc., Nashua NH, USA) from heparin-free serum samples following the manufacturer’s instructions. Results were expressed as values in pg/ml for all tested cytokines for each naturally infected pig and the control group; each sample (serum) was tested neat in duplicate. Healthy pigs were collected from pig farms which had never reported ASFV infection in the Walungu district and, which were ASFV negative by both ELISA and PCR tests.

### Statistical analysis

The relative expression of the target genes for each sample was calculated as described in the previous section and presented as fold changes. One-way analysis of variance (ANOVA) was used to compare the relative concentrations of cytokine proteins and mRNA expression of IFN-α, TNF-α, and IL-10 among groups. Correlation between the mRNA level for each cytokine and the expression of *RelA* mRNA was assessed by the Pearson correlation coefficient test. All statistical analysis was performed using SAS version 9.4 software (SAS Institute Inc., Cary, USA), and a confidence level of 95% was used in all tests to determine the statistical significance between groups. A *p*-value of < 0.05 was considered significant. Visualization and graphical presentations were done using GraphPad Prism 7 software (Diego, CA, USA).

## Results

### Status of African swine fever virus in samples used for assessing RelA polymorphisms

In total, 60 samples were amplified for the *RelA* gene, of which 40 samples resulted in *RelA* sequences of sufficient quality. The origin, clinical assessment, PCR, and ELISA status of these 40 samples are shown in Table 4. As seen, twenty-eight healthy pigs were identified (in cursive), distributed over all 6 districts, and twelve symptomatic pigs were identified with typical ASF symptoms such as cyanotic areas on the skin, fever, difficulties moving, and loss of appetite. This was accompanied by PCR positivity and occasionally positive antibody tests, indicating that they were recently infected. The surviving pigs had all a positive antibody test but a negative PCR test, or were positive in both tests but appeared healthy.

**Table 4:** Origin ASFV, IgG antibody level and PCR status of the samples analyzed

Sample ID	Clinical signs	ELISA	PCR
Fizi (32)	Redness on skin, fever, difficulty moving	+	+
*Fizi (33)*	*Apparently healthy, no sign observed*	+	-
Kalehe (49)	Redness on skin, fever, difficulty moving	+	+
Uvira (50)	Redness on skin, fever, difficulty moving, lack of appetite	-	+
Uvira (53)	Redness on skin, fever, difficulty moving	-	+
Mwenga (57)	Redness on skin, fever, difficulty moving	-	+
Uvira (63)	Redness on skin, fever, difficulty moving	-	+
*Uvira (81)*	*Apparently healthy, no sign observed*	+	-
*Fizi (122)*	*Apparently healthy, no sign observed*	+	-
*Kabare (134)*	*Apparently healthy, no sign observed*	+	-
*Walungu(146)*	*Apparently healthy, no sign observed*	+	-
*Kalehe (226)*	*Apparently healthy, no sign observed*	+	-
*Kabare (230)*	*Apparently healthy, no sign observed*	+	-
*Walungu(238)*	*Apparently healthy, no sign observed*	+	-
*Kalehe (239)*	*Apparently healthy, no sign observed*	+	-
*Walungu (240)*	*Apparently healthy, no sign observed*	+	-
*Walungu (244)*	*Apparently healthy, no sign observed*	+	-
*Kabare (250)*	*Apparently healthy, no sign observed*	+	-
*Kalehe (251)*	*Apparently healthy, no sign observed*	+	-
*Walungu (255)*	*Apparently healthy, no sign observed*	+	-
*Walungu (264)*	*Apparently healthy, no sign observed*	+	-
Walungu (265)	Redness on skin, fever, difficulty moving	+	+
Kalehe (271)	Redness on skin, fever, difficulty moving	+	-
*Kabare (273)*	Redness on skin, fever, difficulty moving	+	-
Kabare (275)	*Apparently healthy, no sign observed*	+	+
*Kabare (276)*	Redness on skin, fever, difficulty moving	+	-
Kabare (277)	*Apparently healthy, no sign observed*	+	+
*Walungu (280)*	*Apparently healthy, no sign observed*	+	-
Walungu (281)	Redness on skin, fever, difficulty moving	-	+
*Uvira (286)*	*Apparently healthy, no sign observed*	+	-
*Uvira (287)*	*Apparently healthy, no sign observed*	+	-
*Uvira (288)*	*Apparently healthy, no sign observed*	+	-
*Uvira (296)*	*Apparently healthy, no sign observed*	+	-
*Fizi (311)*	*Apparently healthy, no sign observed*	+	-
*Walungu (318)*	*Apparently healthy, no sign observed*	+	-
Walungu (326)	Redness on skin, fever, difficulty moving	-	+
*Walungu (329)*	*Apparently healthy, no sign observed*	_+_	-
*Mwenga (331)*	*Apparently healthy, no sign observed*	+	-
*Mwenga (336)*	*Apparently healthy, no sign observed*	+	-
*Kabare (384)*	*Apparently healthy, no sign observed*	+	-

(+): Positive; (-): Negative; low level: (< 1pg/ml); high level: (> 1pg/ml); surviving pigs are in cursive

### A distinct signature in the RelA gene is related to ASFV resistance

Sequence analysis revealed the presence of 28 polymorphisms in the *RelA* (p65) genomic sequence. Of these polymorphic sites, 16 were non-synonymous single nucleotide polymorphisms (nsSNPs) with codon changes resulting in the amino acid changes listed in Table 5.

**Table 5:** The 16 nonsynonymous amino acid differences in the *RelA* gene between the symptomatic and surviving pigs

NO	AA Position	Symptomatic pigs	Surviving pigs	
1	374	P(Proline)	S(Serine)	
2	437	L(Leucine)	P(Proline)	
3	448	T(Threonine)	S(Serine)	
4	455	L(Leucine)	T(Threonine)	
5	460	T(Threonine)	N(Asparagine)	
6	462	P(Proline)	R(Arginine)	
7	464	V(Valine)	Q(Glutamine)	
8	466	T(Threonine)	R(Arginine)	
9	478	Q(Glutamine)	H(Histidine)	
10	491	A(Alaline)	G(Glycine)	
11	495	L(Leucine)	E(Glutamate)	
12	499	P(Proline)	Q(Glutamine)	
13	501	A(Alaline)	G(Glycine)	
14	509	S(Serine)	K(Lysine)	
15	513	P(Proline)	L(Leucine)	
16	517	P(Proline)	T(Threonine)	

**Fig. 3:**
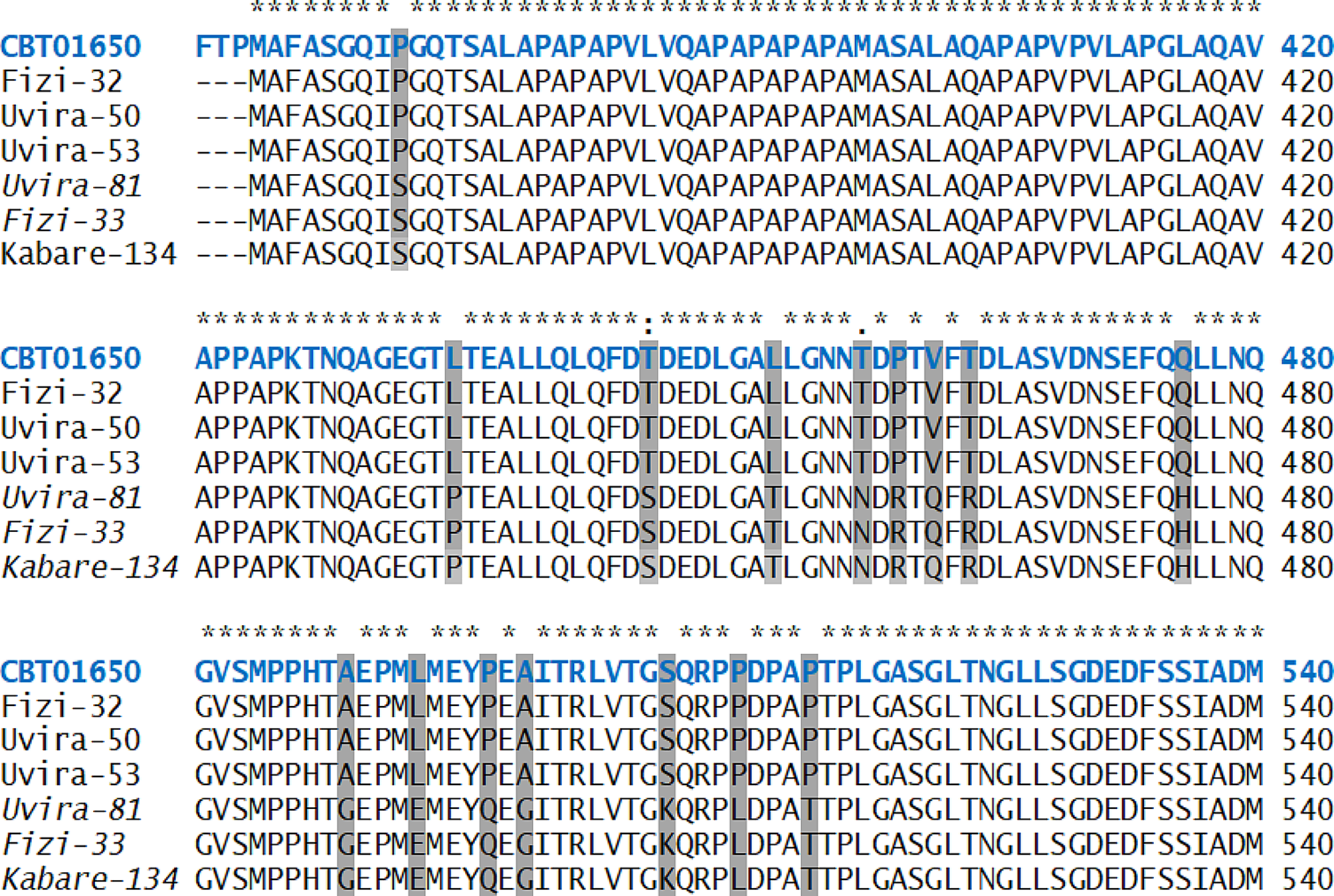
Porcine RelA exon 10 amino acid alignment sequences. Stars (*) in the top indicate conserved ammino acids. The swine RelA reference sequence (Acc. No: CBT01650.1) is in blue. The amino acid variations between the symptomatic and surviving domestic pigs are highlighted. Sample names that are NOT in cursive represent symptomatic (clinical observations) pigs. Sample names in cursive are from surviving pigs

Six representative sequences show this (Fig. 3), whereas all sequences are presented in the supplementary material (Fig. S2). All the nonsynonymous amino acid differences were located in exon 10 within the transactivation domain 2 (TAD2) except one (P374-S) found in the region between TAD2 and nuclear localization signal (NLS). It was stunning to discover that there were only two different protein sequences for *RelA*, one found in surviving pigs and the other in susceptible pigs. Analysis of the nucleotide sequences revealed that this was not due to two discrete alleles, as there were several synonymous nucleotide polymorphisms within these two groups (data not shown). All the identified nonsynonymous SNPs were then subjected to *in silico* analysis to identify possible protein structure and function disturbances.

### Prediction of the effects of SNPs on protein function and phosphorylation sites in the RelA genome sequence using SIFT and PolyPhen software

To resolve the biases associated with predicting effects of SNPs, we combined SIFT [30] and PolyPhen software [31] to predict damaging SNPs. Both software are used to predict whether an amino acid substitution may affect protein function based on sequence homology and the physical properties of amino acids. The programs are described in more detail in M&M. A total of 7 missense nsSNPs were found with a score as either “damaging” (score < 0.05) or “probably damaging” (score > 0.85) in both analyses; these include P374-S, L455-T, P462-R, V464-P, Q478-H, L495-E, and P499-Q. Similarly, two nsSNPs (L437-P) and (A491-G) scored as either “tolerated” (score > 0.05) or “benign” (score < 0.5) in both SIFT and PolyPhen respectively.

However, two (T466-R and S509-K) nsSNPs were predicted to be damaging in SIFT analysis and “possibly damaging” (score > 0.5) in PolyPhen while only one nsSNP (L455-T) was damaging by SIFT, but predicted as benign by PolyPhen (Table 6). The seven amino acid substitutions predicted to be “damaging” and “probably damaging” in both the SIFT and the PolyPhen-2 analyses are highly likely SNPs influencing the structure and function of RelA p65 protein and were therefore considered for further analyses. Analysis of potential phosphorylation sites by NetPhos2.0 revealed that only one site was predicted to undergo phosphorylation with high confidence. This was the serine at position 448 in the surviving pigs (score 0.96), which therefore, could be of critical importance.

**Table 6:** Sequence and structural homology-based prediction of damaging coding nonsynonymous SNPs in the *RelA* gene using SIFT and PolyPhen-2

NO	Substitution	SIFT prediction		PolyPhen-2 prediction	
		Score	prediction	Score	Effect
1	*P374-S*	*0.005*	*Damaging*	*0.999*	*Probably damaging*
2	L437-P	0.11	Tolerated	0.002	Benign
3	*T448-S*	*0.01*	*Damaging*	*0.952*	*Probably damaging*
4	L455T	0.005	Damaging	0.288	Benign
5	T460-N	0.52	Tolerated	0.507	Possibly damaging
6	*P462-R*	*0.01*	*Damaging*	*0.999*	*Probably damaging*
7	*V464-Q*	*0.02*	*Damaging*	*0.985*	*Probably damaging*
8	T466-R	0.005	Damaging	0.791	Possibly damaging
9	*Q478-H*	*0.0*	*Damaging*	*0.998*	*Probably damaging*
10	A491-G	0.06	Tolerated	0.001	Benign
11	*L495-E*	*0.01*	*Damaging*	*0.999*	*Probably damaging*
12	*P499-Q*	*0.0*	*Damaging*	*1.00*	*Probably damaging*
13	A501-G	0.06	Tolerated	0.770	Possibly damaging
14	S509-K	0.0	Damaging	0.770	Possibly damaging
15	P513-L	0.07	Tolerated	0.836	Possibly damaging
16	P517-T	0.21	Tolerated	1.00	Possibly damaging

For SIFT, Scores 0.00 to 0.05: damaging, score, > 0.05: neutral; For PolyPhen-2, score ≥ 0.85: probably damaging, score < 0.5: benign, and 0.5 ≤ score < 0.85): possibly damaging. The 7 nonsynonymous SNP’s effect on protein function is in cursive and scored as either “damaging” or “probably damaging” in both analyses.

### Identification of disease-surviving phenotype associated with nsSNPs using MutPred and predict SNP1

After analysis by MutPred, 5 predicted disease-related amino acid substitutions were found mainly in P374-S, T448-S, P462-R, L495-E, and P499-Q. Among these, amino acid changes such as P374-S, T448-S, and L495-E presenting scores for the probability of pathogenicity of amino acid substitutions (g-value) > 0.50 and *p*-value < 0.05 are referred to as confident hypotheses. The g-values and *p*-values for the 7 amino acid substitutions are presented in Supplementary material Table 1. These SNPs were also predicted as damaging and probably damaging by SIFT and PolyPhen, respectively, with a high score (Table 6). However, P462-R and P499-Q presenting scores with g-value > 0.5 and *p*-value < 0.05 are referred to as actionable hypotheses. Additionally, amino acid substitutions such as V464-Q and Q478-H were predicted not to be associated with disease (g-value < 0.5 and *p*-value > 0.05).

### Effect of amino acid substitutions on mutant protein stability using the MUpro and I-Mutant3 servers

Seven amino acid substitutions predicted to be “probably damaging”using PolyPhen and disease-related using MutPred, were submitted to both the MUPro and the I-Mutant3 servers for prediction of the Delta Delta Gibbs (DDG) stability. The analysis revealed that all 7 mutant amino acids were predicted by the two tools to have a destabilizing effect on the protein (DDG score < 0) (Supplementary material Table 2).

### Evaluation of cytokine protein concentrations in the surviving, the sick (symptomatic), and the healthy (non-infected) pigs

**Fig. 4:**
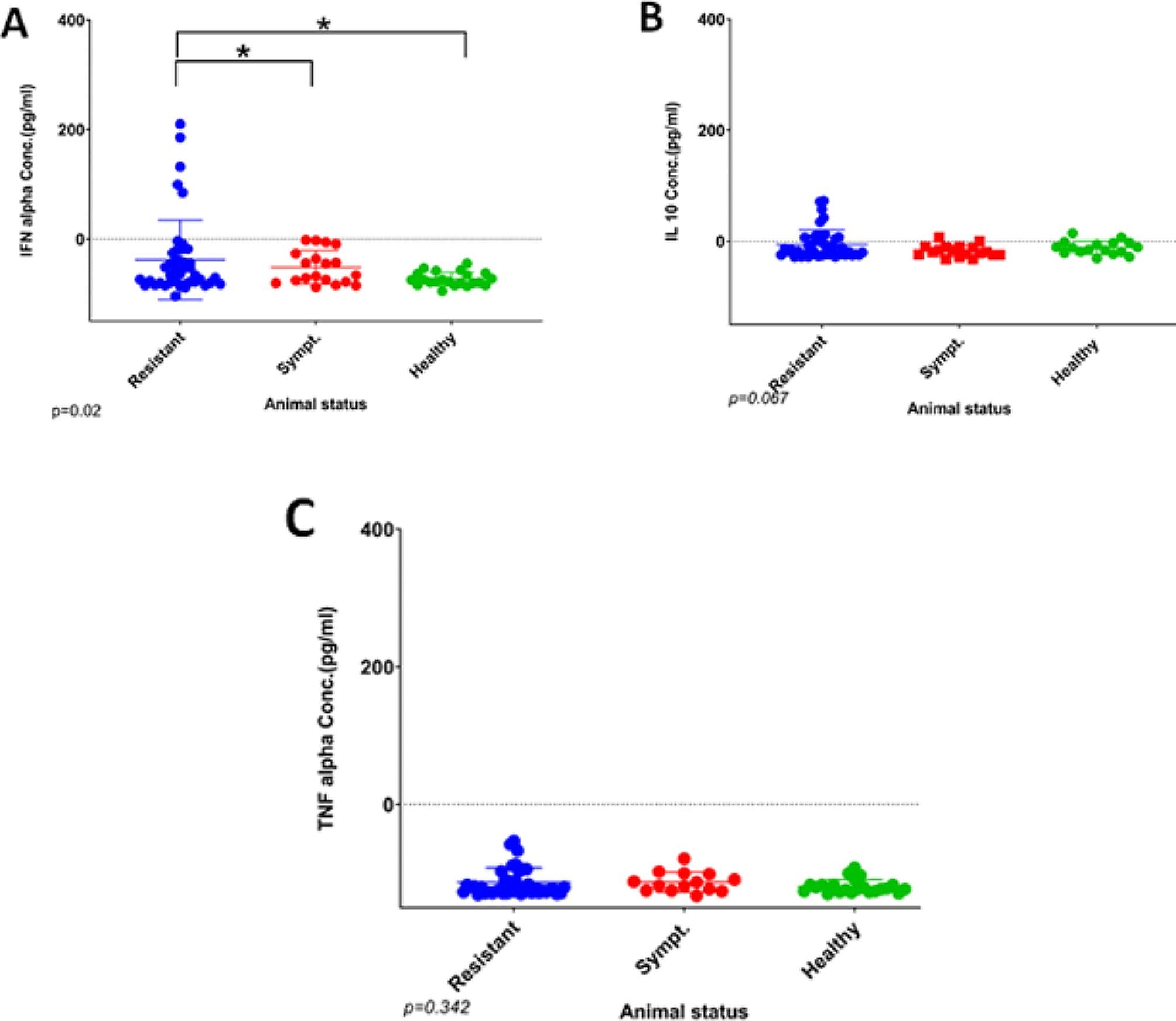
Evaluation of the systemic levels of different host cytokines in farm pigs naturally infected with the ASF virus. Average ratio ± standard error of surviving (blue bars) and symptomatic (red bars) pigs are presented. Values are expressed as a concentration in pg/mL of serum following the instruction of the kit used. IFN-α (**A**), IL-10 (**B**), TNF-α (**C**). Statistically significance (*) with ANOVA test was obtained by comparing surviving to symptomatic and healthy sera

Thirty serum samples from the naturally ASFV-infected pigs and the surviving infected pigs were quantified by ELISA for the cytokines IFN-α, IL-10 and TNF-α. The ELISA results revealed higher levels of IFN-α in the ASFV-surviving pigs compared to the non-infected pigs, showing some pigs with a marked increase compared to susceptible and non-infected pigs. This increase was statistically significant (*P* = 0.02) (Fig. 4A). The susceptible pigs had IFN-α level comparable with the non-infected pigs. Detectable levels of IL-10 were observed in sera from all groups tested, but only with a significant difference between healthy and surviving pigs at the 90% confidence level (*P* = 0.067) (Fig. 4B). For TNF-α, lower values (non-detectable levels, less than zero) were seen in both naturally ASFV-infected and surviving sera (Fig. 4C). Like for the other cytokines, there was a tendency for higher values for some animals in the group of surviving animals. There was a quenching agent in the sera compared to the positive control, which consisted of purified cytokine in PBS that pushed the values below zero. However, in this context, the relative values between groups are of more interest than the exact values, and this quenching effect was similar between groups.

### Quantification of the mRNA expression of IFN-α, IL-10, and TNF-α using real-time PCR

**Fig. 5:**
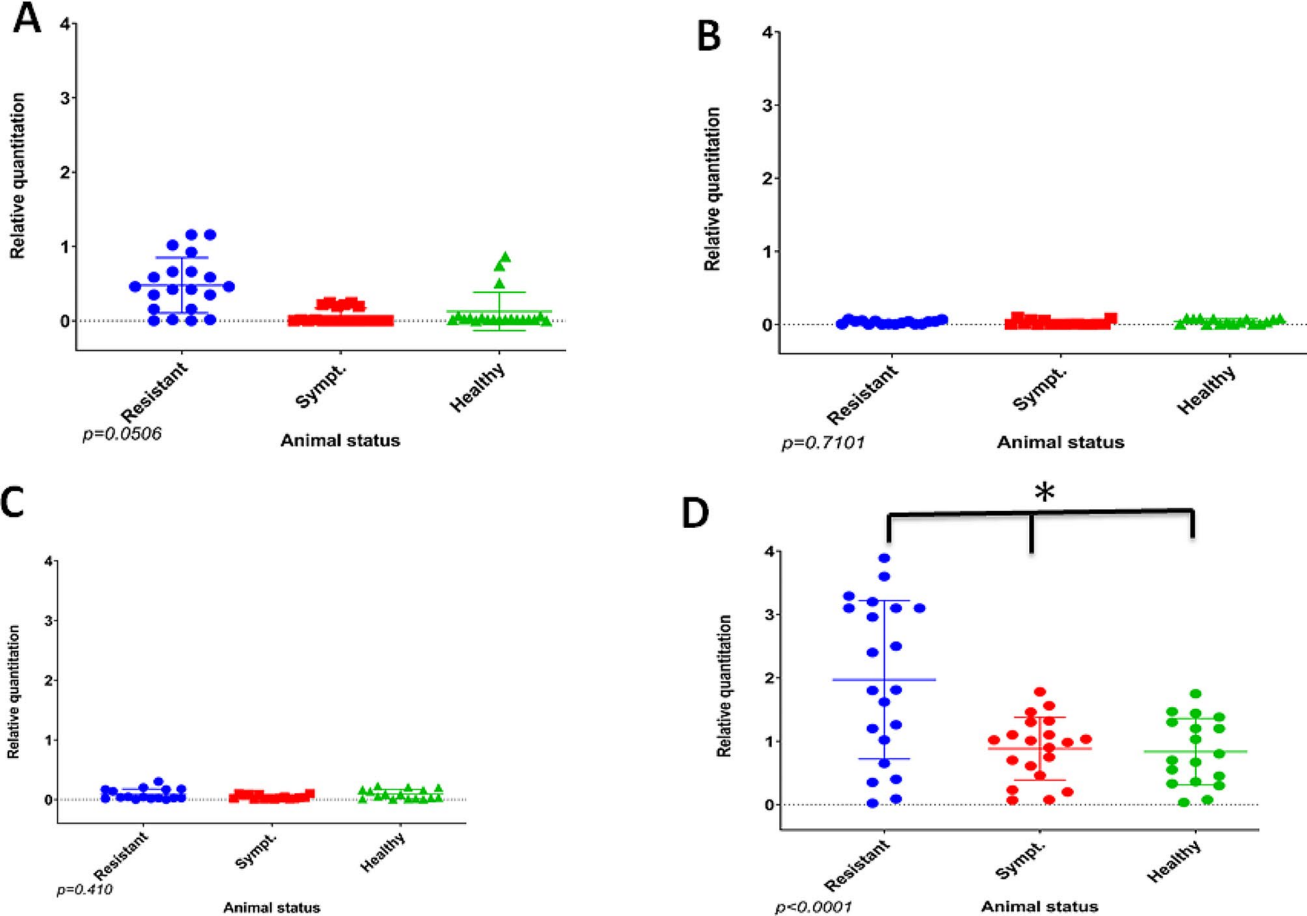
Quantification of mRNA expression of IFN-α (**A**), IL-10 (**B**), TNF-α (**C**), RelA gene (**D**) in blood samples from ASFV surviving, symptomatic, and healthy (non-infected) pigs. Cytokines and RelA from each animal were determined in triple determination. Results are presented as the average group ratio ± standard error (AVG ± SE) between the values of each cytokine normalized with the housekeeping gene 18 S rRNA values. Surviving pigs were compared to symptomatic and healthy pigs

In addition to the ELISA results for the protein expression, real-time PCR was performed to assess the mRNA expression levels of the different cytokines. Results are shown as average group ratios ± standard error (AVG ± SE) between the number of cytokine molecules and the number of molecules of the housekeeping gene 18 S rRNA for the respective sera (Fig. 5). For IFN-α, the qPCR results showed significantly higher levels of mRNA expression in the ASFV-surviving sera relative to the sera from the symptomatic pigs (Fig. 5A). An increase of 0.5x was detected in the ASFV-surviving sera compared to the symptomatic. Additionally, the results showed a significant increase of 30% of IL-10 (*P* = 0.0016) (Fig. 5B) and 10% and TNF-α mRNA expression in the ASFV ASFV-surviving sera compared with sera from symptomatic pigs. However, the differences in expression did not reach statistical significance for TNF-α (Fig. 5C). The porcine RelA (p65) expression in sera from both ASFV-surviving, symptomatic, and healthy pigs were analyzed. Real-time PCR results revealed that the porcine *RelA* (p65) mRNA expression was 2 times higher in surviving pigs compared to symptomatic pigs (*p* < 0.0001) and 2.1 times higher compared to healthy pigs (*p* < 0.0001). No significant differences in expression levels of *RelA* were observed in infected pigs compared to healthy pigs (Fig. 5D).

### Correlation between RelA and cytokine mRNA expression

The Pearson correlation coefficient test was used to assess the correlation between the mRNA levels for each cytokine alongside the expression of *RelA* mRNA. Results of the analysis revealed a statistically significant positive correlation in surviving pigs for IFN-α, (Pears. Coeff. 0.85; 95%CI: 0.28–0.97; *P* = 0.014) and IL-10 (Pears. Coeff. 0.82; 95%CI: 0.19–0.97; *P* = 0.021) with *RelA* mRNA expression, meaning that IFN-α and IL-10 increased expression with the increase of *RelA* mRNA expression in surviving pigs (Table 7). Similarly, the expression of IFN-α (Pears. Coeff. 0.98; 95%CI: 0.92–0.99; *P* < 0.0001) and IL-10 (Pears. Coeff. 0.91; 95%CI: 0.58–0.98; *P* = 0.0015) increased significantly in proportion with the expression of *RelA* mRNA in susceptible pigs (Table 7), but not in healthy uninfected pigs.

**Table 7:** Correlation between the mRNA expression of IFN-α, IL-10 and TNFα with the porcine RelA expression in sick and tolerant pigs

Status	Cytokines	Pearson coefficient	95% CI	P-value
Surviving	IFN-α	0.85	0.28-0.97	0.014**
	IL-10	0.82	0.19-0.97	0.021**
	TNF-α	-0.34	-0.87-0.54	0.444
Susceptibe	IFN-α	0.98	0.92-0.99	<0.0001***
	IL-10	0.91	0.58-0.98	0.0015***
	TNF-α	0.23	-0.56-0.8	0.576
Healthy	IFN-α	0.66	-0.32-0.95	0.153
	IL-10	0.15	-0.74-0.85	0.763
	TNF-α	0.19	-0.73-0.96	0.710

**: statistical significant positive correlation (95%CI), ***: statistical very significant positive correlation (95%CI)

## Discussion

African swine fever virus (ASFV) is a severe hemorrhagic disease associated with huge economic losses in the pig industry worldwide. There is evidence that indigenous pigs in most African countries are asymptomatic carriers of infection from which they rapidly recover [11, 12]. This study utilized a candidate gene sequencing approach to investigate the genetic basis of host immune response to ASFV infection in pigs that had survived ASFV infection and appeared healthy versus pigs that were symptomatic from ASFV infection. The *RelA* gene (expressing p65 protein), one of the key molecules in the NF-kB and nuclear factor of activated T cells (NFAT) host signaling pathways leading to the production of inflammatory cytokines, was sequenced to analyze associations with resistance status in the pigs. In addition, the impact of amino acid substitutions, due to nonsynonymous SNPs, on the stability of the *RelA* protein was assessed, as was the correlation of the *RelA* expression with cytokine levels in the ASFV non-infected and surviving pigs.

In total, 28 sequence differences within the *RelA* gene were found at the genomic level between healthy and surviving domestic pigs. Out of the 28 SNPs identified, 16 were nonsynonymous, resulting in codon change, with the majority being predicted to alter protein function and all of them located in exon 10 within the acidic transactivation domain (TAD2) region, located from the residues 431–554 in porcine p65 protein [39]. In a previous study [40], more than 30 potential polymorphisms were reported in several parts of the porcine *RelA* (p65) gene sequence from China compared with other mammalian species, where polymorphism sites were identified in both RHD and TAD. Among the polymorphisms from this study, two substitution sites, i.e., P374-S and G489-A in surviving pigs corresponded to SNPs in the *RelA* gene at the same positions (P374 and A489), identified in domestic pigs in the study conducted in 2011 [40]. Additionally, one substitution, S448-T corresponded to an SNP identified in a previous study conducted by Palgrave et al. [10], which was predicted to change the protein structure by conferring hydrophobicity in the domestic pig sequence compared to the warthog. Some amino acid residues, i.e., serine 536 and serine 276 (in TAD), have been related to known functions of *RelA*, such as phosphorylation [41]. Comparative analysis of the *RelA* homology domain (RHL) and the nuclear localization signal in RelA showed complete sequence similarity in symptomatic, surviving, and non-infected (healthy) pigs (data not shown). In the present study, one of the identified nonsynonymous amino acid substitutions was (P374S), which lies in the TAD1 region of p65, while the other 15 were located in the TAD2. In a previous study aiming at revealing signatures for resistance to ASF, genetic variations were identified in both TAD1 and TAD2 of the *RelA* subunit of NF-kB of the domestic pig compared with warthog [10]. TAD1 and TAD2 are involved mainly in activating the transcription of p65-regulated genes, and it operates by participating in a series of protein–protein interactions with various transcriptional regulatory proteins [39]. Based on this, variations within this region could play a significant role in the pathogenicity of ASFV.

Of the 16 nonsynonymous SNPs found, one (serine at position 448) was predicted to be a phosphorylation site in the surviving group and is located within the TAD2. This contrasts with the previous study that has identified a phosphorylation site at serine-531 within the TAD1 of the domestic pig *RelA* gene [10]. The threonine at position 448 in the symptomatic domestic pig from this study is substituted by a serine residue in the surviving pigs, whereas the threonine was found to be substituted for an alanine residue in the warthog [10]. The limited availability of porcine *RelA* sequence data, however, makes it difficult to make a conclusive comparative analysis of this site. Since up to one-third of eukaryotic proteome functions are controlled by phosphorylation [42], the present polymorphism at position 448 could play a prominent role in defining the host response to ASFV infection in domestic pigs. This is supported by a previous finding showing that the phosphorylation of RelA induces a structural change, which impacts its ubiquitination and stability, as well as protein-protein interactions [43].

The damaging/deleterious prediction scores by a combination of sequence and structural homology-based tools, SIFT and PolyPhen-2, revealed respectively 10/16 (62.5%) and 13/16 (81.2%) of nsSNPs as damaging/deleterious. The results of MutPred and PredictSNP predicted 5 high-confidence nsSNPs, i.e. P374-S, T448-S, P462-R, L495-E, and P449-Q, which are associated with the symptomatic phenotype, as damaging. However, the MUPro and I-Mutant3 predictions servers demonstrated seven high-confidence nsSNPs that affect protein stability, and these SNPs were predicted to be damaging/deleterious in both SIFT and Polyphen-2. As these SNPs all occurred in the same sequence resulting in amino acid change, this will likely influence the function of *RelA*, and as such lead to a difference in *RelA* function in surviving versus symptomatic pigs.

The qPCR and ELISA showed a significant increase of IFN-α transcripts and cytokine expression levels in ASFV- surviving infected pigs compared to symptomatic and healthy pigs. The difference between susceptible and surviving pigs could be attributed to differences in the host responses to ASFV infection. The stimulated Toll-Like Receptors (TLRs), after infection by a pathogen, induce the activation of signal transduction cascades, which oblige the nuclear factor-κB (NF-κB) to translocate to the nucleus [44], followed by activation of interferon regulatory factors 3/7 (IRF3/7) or activator protein-1 (AP-1), which cooperate to initiate transcription of different cytokines such as alpha/beta interferon (IFN-α/β) to counteract infection [45, 46]. The pathways leading to Type 1 interferons and proinflammatory cytokines such as TNF alpha, IL6, and IL12 have traditionally been viewed as relatively discrete pathways. However, it has become evident that there is considerable cross-talk between the NF-kB and the IRF pathways [47, 48]. Previous investigation on NF-κB-deficient cells has revealed that type I interferon (IFN) response relies mainly on concurrent NF-κB activation [49]. In addition, an experimental assay has demonstrated that in the absence of NF-κB, the rapid expression of IFNβ is blunted, reducing the propagation of anti-viral signals in the mucosal surface [50]. Moreover, NF-κB also controls the expression of the downstream IFN auto-amplification loop through STAT1, IRF-1, − 5, and − 7 transcription factors. This points to NF-kB as a central regulator of the combined pro-inflammatory response. Therefore, a distortion of the activity of NF-kB due to SNPs in *RelA* may lead to a lower response of type I interferons, as we observed in the susceptible group of pigs. In addition to this, we also observed a higher *RelA* expression in the surviving pigs, which can also contribute to the findings of the increased systemic IFN levels seen in these pigs.

Interestingly, there was an increase in both the gene expression and protein levels of IL-10 in the surviving pigs. IL10 is generally an anti-inflamatory cytokine that works to dampen the immune response and avoid tissue damage after initially high levels of proinflammatory cytokines such as TNF-alpha, IL6 and IL12 [15]. It could be a vital element in coping with ASF infection, to dampen the immune system and thereby damage to the body [51]. In contrast, *TNF-α* gene expression and the corresponding protein levels were moderate in both surviving and non-infected pigs, with a tendency to increased levels in the surviving pigs. This is likely due to the timing of samples as TNF-alpha is one of the very first pro-inflammatory cytokines to appear after infection, but it has also been found that TNF-α mRNA and other pro-inflammatory cytokines were inhibited by ASFV infection, both in vitro and in vivo in wild boars [52, 53]. Such inhibition could be due to anti-inflammatory signals by, e.g., IL10, as it has previously been shown that IL10 directly inhibits NF-κB activity and, thereby, the expression of pro-inflammatory cytokines [54].

The association analysis revealed a positive correlation between porcine *RelA* (p65) gene mRNA expression and the mRNA expression level of IFN-α and IL-10 cytokines. This supports the important role of NF-kB in the interferon pathway and suggests substantial crosstalk between the pathways. The positive correlation between RelA and IL10 may at first glance appear strange, as IL10 suppresses the NF-kB activity, but IL10 exerts its inhibition in an RelA-independent manner, acting at least partly through inhibition of translocation of RelA to the nucleus but allowing translocation of the p50 homodimer, which has no transactivating domain. The homodimer binds to the DNA and inhibits the binding of functional NF-kB and the transcription of pro-inflammatory cytokines [54]. Therefore, the positive correlation between NF-kB and IL10 may be initiated by higher activity of NF-KB, leading to increased pro-inflammatory cytokines, then, in turn, an increased IL10 production. The positive correlations between *RelA* mRNA and IFN-α and IL-10 suggest a higher overall activity of the *RelA* subunit of NF-kB in surviving pigs compared to symptomatic pigs, which may be due to the polymorphisms found in the surviving version of the *RelA* gene. The SNPs found in the surviving pigs were all in exon 10, the transactivating domain (TAD2), except one (S9P), which was found in the region between TAD2 and nuclear localization signal (NLS). The TAD2 polymorphisms could affect the efficiency of the viral NF-kBinhibitors, such as the ASFV A238L protein known to have 40% homology to porcine NF-kB inhibitor IkBα, and which binds directly to the *RelA* (p65) subunit of NF-kB to inactivate NF-kB [52]. The A238L protein has also been reported to act as a potential immunosuppressant by inhibition of transcriptional activation from the TNF-α promoter through a mechanism that involves the CREB-binding protein (CBP) transcriptional co-activators [25, 52].

A previous study found lower polymorphisms in the *RelA* subunit of NF-kB from warthogs, which are surviving to ASFV infection [10] than we found in our pig study. They speculated that the three SNP’ found in the warthog gene (compared to pigs) could be responsible for the surviving status seen in warthogs. A transgenic pig was made using the warthog *RelA* gene, but it was found that the pigs were not surviving to ASF. However, a delay in the onset of clinical signs and less viral DNA in blood samples and nasal secretions was observed in some animals [55]. However, the mechanism behind tolerance in domestic pigs may not be identical to what is found in warthogs.

Nevertheless, it is striking that one distinct amino acid sequence of *RelA* is present in the surviving pigs in this study, and the two variant RelA proteins seem to be stable versions of the protein as there were not found any other variants at the amino acid level but several variants at the nucleotide level. An effect of the Sanger sequencing in the present study is that pigs that are heterozygous for the RelA gene will have been discarded in the process due to non-conclusive sequences, leaving only the homozygous pigs left for the association, but this is likely to contribute to the highly conclusive finding. We hypothesize that these SNPs (found in the surviving animals) lead to an escape from ASFV suppression, e.g., by the virus gene A238L, which then leads to an increased NF-kB activity, higher cytokine profiles, and resistance of the pigs. One limitation of the study is the lack of knowledge of the ASFV strain(s) in sampling areas. There could have been more than one strain/genotype circulating, and there could have been differences in the virulence of these strains, explaining why some animals survive the infection and others do not. Still, this would not explain the association between the RelA sequence and phenotype found in this study. Further investigation particularly an in silico study is warranted in order to identify which domains are modified and the protein function by the observed amino acid modifications on RelA gene.

### Electronic supplementary material

Below is the link to the electronic supplementary material.

**Supplementary Material 1: Fig S1:** The NF-κB family

**Supplementary Material 2: Fig S2:** Porcine RelA exon 10 amino acid alignment sequences

**Supplementary Material 3: Table S1:** Prediction of disease-related amino acid substitutions by MutPred and PredictSNP sofwares

**Supplementary Material 4: Table S2:** Prediction of free energy change using MUPro andI-Mutant3 servers

## Data Availability

The datasets supporting the conclusions of this article are included within the article and its Supplementary Materials, further inquiries can be directed to the corresponding author, Patrick Bisimwa (patrick.ntagereka@gmail.com).
