# Supplementary material for: Resistance to African swine fever virus among African domestic pigs appears to be associated with a distinct polymorphic signature in the RelA gene and upregulation of RelA transcription
Source: Virol J. 2024 Apr 24;21:93. doi: 10.1186/s12985-024-02351-9 (PMC11041040; doi:10.1186/s12985-024-02351-9)
Supplement: Supplementary file 1 — Supplementary Material 1: Fig S1: The NF-κB family [file 12985_2024_2351_MOESM1_ESM.docx]

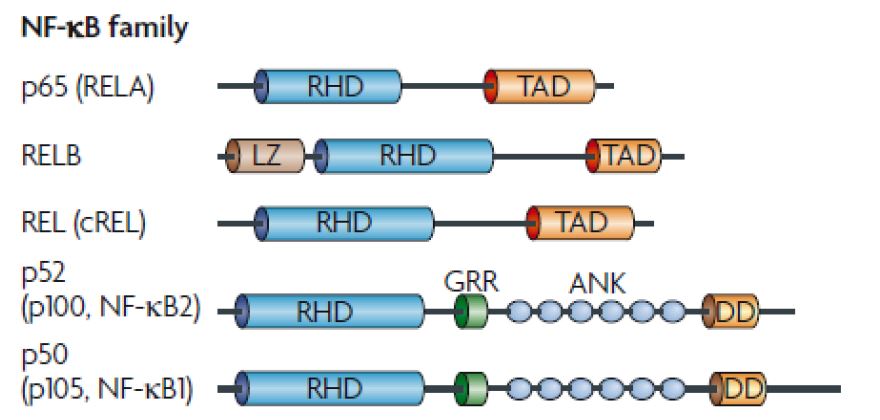


**Supplemenatry material Figure1: The NF-κB family.** This family consists of five members such as, RelA, RelB, cRel, p50 and p52. The RHD is conserved among all of these members and mediates DNA binding, dimerization and nuclear localization. The TAD, is present only in RelA, RelB and cRel which allows transactivation of gene expression (Ghosh and Hayden, 2008).
