# Supplementary material for: Resistance to African swine fever virus among African domestic pigs appears to be associated with a distinct polymorphic signature in the RelA gene and upregulation of RelA transcription
Source: Virol J. 2024 Apr 24;21:93. doi: 10.1186/s12985-024-02351-9 (PMC11041040; doi:10.1186/s12985-024-02351-9)
Supplement: Supplementary file 2 — Supplementary Material 2: Fig S2: Porcine RelA exon 10 amino acid alignment sequences [file 12985_2024_2351_MOESM2_ESM.docx]

******** ************************************************

CBT01650.1 FTPMAFASGQIPGQTSALAPAPAPVLVQAPAPAPAPAMASALAQAPAPVPVLAPGLAQAV 420

**Fizi-32** ---MAFASGQIPGQTSALAPAPAPVLVQAPAPAPAPAMASALAQAPAPVPVLAPGLAQAV 420

**Uvira-50** ---MAFASGQIPGQTSALAPAPAPVLVQAPAPAPAPAMASALAQAPAPVPVLAPGLAQAV 420

**Kalehe-49** ---MAFASGQIPGQTSALAPAPAPVLVQAPAPAPAPAMASALAQAPAPVPVLAPGLAQAV 420

**Uvira-53** ---MAFASGQIPGQTSALAPAPAPVLVQAPAPAPAPAMASALAQAPAPVPVLAPGLAQAV 420

**Mwenga-57** ---MAFASGQIPGQTSALAPAPAPVLVQAPAPAPAPAMASALAQAPAPVPVLAPGLAQAV 420

**Uvira-63** ---MAFASGQIPGQTSALAPAPAPVLVQAPAPAPAPAMASALAQAPAPVPVLAPGLAQAV 420

**Walungu-265** ---MAFASGQIPGQTSALAPAPAPVLVQAPAPAPAPAMASALAQAPAPVPVLAPGLAQAV 420

**Kalehe-271** ---MAFASGQIPGQTSALAPAPAPVLVQAPAPAPAPAMASALAQAPAPVPVLAPGLAQAV 420

Kabare-273 ---MAFASGQIPGQTSALAPAPAPVLVQAPAPAPAPAMASALAQAPAPVPVLAPGLAQAV 420

Kabare-275 ---MAFASGQIPGQTSALAPAPAPVLVQAPAPAPAPAMASALAQAPAPVPVLAPGLAQAV 420

**Kabare-276** ---MAFASGQIPGQTSALAPAPAPVLVQAPAPAPAPAMASALAQAPAPVPVLAPGLAQAV 420

**Kabare-277** ---MAFASGQIPGQTSALAPAPAPVLVQAPAPAPAPAMASALAQAPAPVPVLAPGLAQAV 420

**Walungu-281** ---MAFASGQIPGQTSALAPAPAPVLVQAPAPAPAPAMASALAQAPAPVPVLAPGLAQAV 420

**Walungu-326** ---MAFASGQIPGQTSALAPAPAPVLVQAPAPAPAPAMASALAQAPAPVPVLAPGLAQAV 420

Fizi-33 ---MAFASGQISGQTSALAPAPAPVLVQAPAPAPAPAMASALAQAPAPVPVLAPGLAQAV 420

Uvira-81 ---MAFASGQISGQTSALAPAPAPVLVQAPAPAPAPAMASALAQAPAPVPVLAPGLAQAV 420

Fizi-122 ---MAFASGQISGQTSALAPAPAPVLVQAPAPAPAPAMASALAQAPAPVPVLAPGLAQAV 420

Kabare-134 ---MAFASGQISGQTSALAPAPAPVLVQAPAPAPAPAMASALAQAPAPVPVLAPGLAQAV 420

Walungu-146 ---MAFASGQISGQTSALAPAPAPVLVQAPAPAPAPAMASALAQAPAPVPVLAPGLAQAV 420

Kalehe-226 ---MAFASGQISGQTSALAPAPAPVLVQAPAPAPAPAMASALAQAPAPVPVLAPGLAQAV 420

Kabare-230 ---MAFASGQISGQTSALAPAPAPVLVQAPAPAPAPAMASALAQAPAPVPVLAPGLAQAV 420

Walungu-238 ---MAFASGQISGQTSALAPAPAPVLVQAPAPAPAPAMASALAQAPAPVPVLAPGLAQAV 420

Kalehe-239 ---MAFASGQISGQTSALAPAPAPVLVQAPAPAPAPAMASALAQAPAPVPVLAPGLAQAV 420

Walungu-240 ---MAFASGQISGQTSALAPAPAPVLVQAPAPAPAPAMASALAQAPAPVPVLAPGLAQAV 420

Walungu-244 ---MAFASGQISGQTSALAPAPAPVLVQAPAPAPAPAMASALAQAPAPVPVLAPGLAQAV 420

Kabare-250 ---MAFASGQISGQTSALAPAPAPVLVQAPAPAPAPAMASALAQAPAPVPVLAPGLAQAV 420

Kalehe-251 ---MAFASGQISGQTSALAPAPAPVLVQAPAPAPAPAMASALAQAPAPVPVLAPGLAQAV 420

Walungu-255 ---MAFASGQISGQTSALAPAPAPVLVQAPAPAPAPAMASALAQAPAPVPVLAPGLAQAV 420

Kabare-277 ---MAFASGQISGQTSALAPAPAPVLVQAPAPAPAPAMASALAQAPAPVPVLAPGLAQAV 420

Walungu-264 ---MAFASGQISGQTSALAPAPAPVLVQAPAPAPAPAMASALAQAPAPVPVLAPGLAQAV 420

Walungu-280 ---MAFASGQISGQTSALAPAPAPVLVQAPAPAPAPAMASALAQAPAPVPVLAPGLAQAV 420

Uvira-286 ---MAFASGQISGQTSALAPAPAPVLVQAPAPAPAPAMASALAQAPAPVPVLAPGLAQAV 420

Uvira-287 ---MAFASGQISGQTSALAPAPAPVLVQAPAPAPAPAMASALAQAPAPVPVLAPGLAQAV 420

Uvira-288 ---MAFASGQISGQTSALAPAPAPVLVQAPAPAPAPAMASALAQAPAPVPVLAPGLAQAV 420

Fizi-296 ---MAFASGQISGQTSALAPAPAPVLVQAPAPAPAPAMASALAQAPAPVPVLAPGLAQAV 420

Fizi-311 ---MAFASGQISGQTSALAPAPAPVLVQAPAPAPAPAMASALAQAPAPVPVLAPGLAQAV 420

Walungu-318 ---MAFASGQISGQTSALAPAPAPVLVQAPAPAPAPAMASALAQAPAPVPVLAPGLAQAV 420

Walungu-329 ---MAFASGQISGQTSALAPAPAPVLVQAPAPAPAPAMASALAQAPAPVPVLAPGLAQAV 420

Mwenga-331 ---MAFASGQISGQTSALAPAPAPVLVQAPAPAPAPAMASALAQAPAPVPVLAPGLAQAV 420

Mwenga-336 ---MAFASGQISGQTSALAPAPAPVLVQAPAPAPAPAMASALAQAPAPVPVLAPGLAQAV 420

Kabare-384 ---MAFASGQISGQTSALAPAPAPVLVQAPAPAPAPAMASALAQAPAPVPVLAPGLAQAV 420

************** **********:****** **** * * * *********** ****

CBT01650.1 APPAPKTNQAGEGTLTEALLQLQFDTDEDLGALLGNNTDPTVFTDLASVDNSEFQQLLNQ 480

**Fizi-32** APPAPKTNQAGEGTLTEALLQLQFDTDEDLGALLGNNTDPTVFTDLASVDNSEFQQLLNQ 480

**Kalehe-49** APPAPKTNQAGEGTLTEALLQLQFDTDEDLGALLGNNTDPTVFTDLASVDNSEFQQLLNQ 480

**Uvira-50** APPAPKTNQAGEGTLTEALLQLQFDTDEDLGALLGNNTDPTVFTDLASVDNSEFQQLLNQ 480

**Uvira-53** APPAPKTNQAGEGTLTEALLQLQFDTDEDLGALLGNNTDPTVFTDLASVDNSEFQQLLNQ 480

**Mwenga-57** APPAPKTNQAGEGTLTEALLQLQFDTDEDLGALLGNNTDPTVFTDLASVDNSEFQQLLNQ 480

**Uvira-63** APPAPKTNQAGEGTLTEALLQLQFDTDEDLGALLGNNTDPTVFTDLASVDNSEFQQLLNQ 480

**Kabare-265** APPAPKTNQAGEGTLTEALLQLQFDTDEDLGALLGNNTDPTVFTDLASVDNSEFQQLLNQ 480

**Walungu-271** APPAPKTNQAGEGTLTEALLQLQFDTDEDLGALLGNNTDPTVFTDLASVDNSEFQQLLNQ 480

Kalehe-273 APPAPKTNQAGEGTLTEALLQLQFDTDEDLGALLGNNTDPTVFTDLASVDNSEFQQLLNQ 480

Kabare-275 APPAPKTNQAGEGTLTEALLQLQFDTDEDLGALLGNNTDPTVFTDLASVDNSEFQQLLNQ 480

**Kabare-276** APPAPKTNQAGEGTLTEALLQLQFDTDEDLGALLGNNTDPTVFTDLASVDNSEFQQLLNQ 480

**Kabare-277** APPAPKTNQAGEGTLTEALLQLQFDTDEDLGALLGNNTDPTVFTDLASVDNSEFQQLLNQ 480

**Walungu-281** APPAPKTNQAGEGTLTEALLQLQFDTDEDLGALLGNNTDPTVFTDLASVDNSEFQQLLNQ 480

**Walungu-326** APPAPKTNQAGEGTLTEALLQLQFDTDEDLGALLGNNTDPTVFTDLASVDNSEFQQLLNQ 480

Fizi-33 APPAPKTNQAGEGTPTEALLQLQFDSDEDLGATLGNNNDRTQFRDLASVDNSEFQHLLNQ 480

Uvira-81 APPAPKTNQAGEGTPTEALLQLQFDSDEDLGATLGNNNDRTQFRDLASVDNSEFQHLLNQ 480

Fizi-122 APPAPKTNQAGEGTPTEALLQLQFDSDEDLGATLGNNNDRTQFRDLASVDNSEFQHLLNQ 480

Kabare-134 APPAPKTNQAGEGTPTEALLQLQFDSDEDLGATLGNNNDRTQFRDLASVDNSEFQHLLNQ 480

Walungu-146 APPAPKTNQAGEGTPTEALLQLQFDSDEDLGATLGNNNDRTQFRDLASVDNSEFQHLLNQ 480

Kalehe-226 APPAPKTNQAGEGTPTEALLQLQFDSDEDLGATLGNNNDRTQFRDLASVDNSEFQHLLNQ 480

Kabare-230 APPAPKTNQAGEGTPTEALLQLQFDSDEDLGATLGNNNDRTQFRDLASVDNSEFQHLLNQ 480

Walungu-238 APPAPKTNQAGEGTPTEALLQLQFDSDEDLGATLGNNNDRTQFRDLASVDNSEFQHLLNQ 480

Kalehe-239 APPAPKTNQAGEGTPTEALLQLQFDSDEDLGATLGNNNDRTQFRDLASVDNSEFQHLLNQ 480

Walungu-240 APPAPKTNQAGEGTPTEALLQLQFDSDEDLGATLGNNNDRTQFRDLASVDNSEFQHLLNQ 480

Walungu-244 APPAPKTNQAGEGTPTEALLQLQFDSDEDLGATLGNNNDRTQFRDLASVDNSEFQHLLNQ 480

Kabare-250 APPAPKTNQAGEGTPTEALLQLQFDSDEDLGATLGNNNDRTQFRDLASVDNSEFQHLLNQ 480

Kalehe-251 APPAPKTNQAGEGTPTEALLQLQFDSDEDLGATLGNNNDRTQFRDLASVDNSEFQHLLNQ 480

Walungu-255 APPAPKTNQAGEGTPTEALLQLQFDSDEDLGATLGNNNDRTQFRDLASVDNSEFQHLLNQ 480

Walungu-264 APPAPKTNQAGEGTPTEALLQLQFDSDEDLGATLGNNNDRTQFRDLASVDNSEFQHLLNQ 480

Walungu-280 APPAPKTNQAGEGTPTEALLQLQFDSDEDLGATLGNNNDRTQFRDLASVDNSEFQHLLNQ 480

Uvira-286 APPAPKTNQAGEGTPTEALLQLQFDSDEDLGATLGNNNDRTQFRDLASVDNSEFQHLLNQ 480

Uvira-287 APPAPKTNQAGEGTPTEALLQLQFDSDEDLGATLGNNNDRTQFRDLASVDNSEFQHLLNQ 480

Uvira-288 APPAPKTNQAGEGTPTEALLQLQFDSDEDLGATLGNNNDRTQFRDLASVDNSEFQHLLNQ 480

Uvira-296 APPAPKTNQAGEGTPTEALLQLQFDSDEDLGATLGNNNDRTQFRDLASVDNSEFQHLLNQ 480

Fizi-311 APPAPKTNQAGEGTPTEALLQLQFDSDEDLGATLGNNNDRTQFRDLASVDNSEFQHLLNQ 480

Fizi-318 APPAPKTNQAGEGTPTEALLQLQFDSDEDLGATLGNNNDRTQFRDLASVDNSEFQHLLNQ 480

Walungu-329 APPAPKTNQAGEGTPTEALLQLQFDSDEDLGATLGNNNDRTQFRDLASVDNSEFQHLLNQ 480

Mwenga-331 APPAPKTNQAGEGTPTEALLQLQFDSDEDLGATLGNNNDRTQFRDLASVDNSEFQHLLNQ 480

Mwenga-336 APPAPKTNQAGEGTPTEALLQLQFDSDEDLGATLGNNNDRTQFRDLASVDNSEFQHLLNQ 480

Kabare-384 APPAPKTNQAGEGTPTEALLQLQFDSDEDLGATLGNNNDRTQFRDLASVDNSEFQHLLNQ 480

******** *** *** * ******* *** *** *************************

CBT01650.1 GVSMPPHTAEPMLMEYPEAITRLVTGSQRPPDPAPTPLGASGLTNGLLSGDEDFSSIADM 540

**Fizi-32** GVSMPPHTAEPMLMEYPEAITRLVTGSQRPPDPAPTPLGASGLTNGLLSGDEDFSSIADM 540

**Kalehe-49** GVSMPPHTAEPMLMEYPEAITRLVTGSQRPPDPAPTPLGASGLTNGLLSGDEDFSSIADM 540

**Uvira-50** GVSMPPHTAEPMLMEYPEAITRLVTGSQRPPDPAPTPLGASGLTNGLLSGDEDFSSIADM 540

**Uvira-53** GVSMPPHTAEPMLMEYPEAITRLVTGSQRPPDPAPTPLGASGLTNGLLSGDEDFSSIADM 540

**Mwenga-57** GVSMPPHTAEPMLMEYPEAITRLVTGSQRPPDPAPTPLGASGLTNGLLSGDEDFSSIADM 540

**Uvira-63** GVSMPPHTAEPMLMEYPEAITRLVTGSQRPPDPAPTPLGASGLTNGLLSGDEDFSSIADM 540

**Walungu-265** GVSMPPHTAEPMLMEYPEAITRLVTGSQRPPDPAPTPLGASGLTNGLLSGDEDFSSIADM 540

**Kalehe-271** GVSMPPHTAEPMLMEYPEAITRLVTGSQRPPDPAPTPLGASGLTNGLLSGDEDFSSIADM 540

Kabare-273 GVSMPPHTAEPMLMEYPEAITRLVTGSQRPPDPAPTPLGASGLTNGLLSGDEDFSSIADM 540

Kabare-275 GVSMPPHTAEPMLMEYPEAITRLVTGSQRPPDPAPTPLGASGLTNGLLSGDEDFSSIADM 540

**Kabare-276** GVSMPPHTAEPMLMEYPEAITRLVTGSQRPPDPAPTPLGASGLTNGLLSGDEDFSSIADM 540

**Kabare-277** GVSMPPHTAEPMLMEYPEAITRLVTGSQRPPDPAPTPLGASGLTNGLLSGDEDFSSIADM 540

**Walungu-281** GVSMPPHTAEPMLMEYPEAITRLVTGSQRPPDPAPTPLGASGLTNGLLSGDEDFSSIADM 540

**Walungu-326** GVSMPPHTAEPMLMEYPEAITRLVTGSQRPPDPAPTPLGASGLTNGLLSGDEDFSSIADM 540

Fizi-33 GVSMPPHTGEPMEMEYQEGITRLVTGKQRPLDPATTPLGASGLTNGLLSGDEDFSSIADM 540

Uvira-81 GVSMPPHTGEPMEMEYQEGITRLVTGKQRPLDPATTPLGASGLTNGLLSGDEDFSSIADM 540

Fizi-122 GVSMPPHTGEPMEMEYQEGITRLVTGKQRPLDPATTPLGASGLTNGLLSGDEDFSSIADM 540

Kabare-134 GVSMPPHTGEPMEMEYQEGITRLVTGKQRPLDPATTPLGASGLTNGLLSGDEDFSSIADM 540

Walungu-146 GVSMPPHTGEPMEMEYQEGITRLVTGKQRPLDPATTPLGASGLTNGLLSGDEDFSSIADM 540

Kalehe-226 GVSMPPHTGEPMEMEYQEGITRLVTGKQRPLDPATTPLGASGLTNGLLSGDEDFSSIADM 540

Kabare-230 GVSMPPHTGEPMEMEYQEGITRLVTGKQRPLDPATTPLGASGLTNGLLSGDEDFSSIADM 540

Walungu-238 GVSMPPHTGEPMEMEYQEGITRLVTGKQRPLDPATTPLGASGLTNGLLSGDEDFSSIADM 540

Kalehe-239 GVSMPPHTGEPMEMEYQEGITRLVTGKQRPLDPATTPLGASGLTNGLLSGDEDFSSIADM 540

Walungu-240 GVSMPPHTGEPMEMEYQEGITRLVTGKQRPLDPATTPLGASGLTNGLLSGDEDFSSIADM 540

Walungu-244 GVSMPPHTGEPMEMEYQEGITRLVTGKQRPLDPATTPLGASGLTNGLLSGDEDFSSIADM 540

Kabare-250 GVSMPPHTGEPMEMEYQEGITRLVTGKQRPLDPATTPLGASGLTNGLLSGDEDFSSIADM 540

Kalehe-251 GVSMPPHTGEPMEMEYQEGITRLVTGKQRPLDPATTPLGASGLTNGLLSGDEDFSSIADM 540

Walungu-255 GVSMPPHTGEPMEMEYQEGITRLVTGKQRPLDPATTPLGASGLTNGLLSGDEDFSSIADM 540

Walungu-264 GVSMPPHTGEPMEMEYQEGITRLVTGKQRPLDPATTPLGASGLTNGLLSGDEDFSSIADM 540

Walungu-280 GVSMPPHTGEPMEMEYQEGITRLVTGKQRPLDPATTPLGASGLTNGLLSGDEDFSSIADM 540

Uvira-286 GVSMPPHTGEPMEMEYQEGITRLVTGKQRPLDPATTPLGASGLTNGLLSGDEDFSSIADM 540

Uvira-287 GVSMPPHTGEPMEMEYQEGITRLVTGKQRPLDPATTPLGASGLTNGLLSGDEDFSSIADM 540

Uvira-288 GVSMPPHTGEPMEMEYQEGITRLVTGKQRPLDPATTPLGASGLTNGLLSGDEDFSSIADM 540

Uvira-296 GVSMPPHTGEPMEMEYQEGITRLVTGKQRPLDPATTPLGASGLTNGLLSGDEDFSSIADM 540

Fizi-311 GVSMPPHTGEPMEMEYQEGITRLVTGKQRPLDPATTPLGASGLTNGLLSGDEDFSSIADM 540

Fizi-318 GVSMPPHTGEPMEMEYQEGITRLVTGKQRPLDPATTPLGASGLTNGLLSGDEDFSSIADM 540

Walungu-329 GVSMPPHTGEPMEMEYQEGITRLVTGKQRPLDPATTPLGASGLTNGLLSGDEDFSSIADM 540

Mwenga-331 GVSMPPHTGEPMEMEYQEGITRLVTGKQRPLDPATTPLGASGLTNGLLSGDEDFSSIADM 540

Mwenga-336 GVSMPPHTGEPMEMEYQEGITRLVTGKQRPLDPATTPLGASGLTNGLLSGDEDFSSIADM 540

Kabare-384 GVSMPPHTGEPMEMEYQEGITRLVTGKQRPLDPATTPLGASGLTNGLLSGDEDFSSIADM 540

**Supplementary material Figure 2.** Porcine *RelA* exon 10 amino acid alignment sequences. Dark stars (*) in the top indicate conserved sequence motifs. The amino acid variations between the symptomatic and asymptomatic domestic pig are highlighted. The *RelA* reference sequence is in blue color. Sample names which are bold represent symptomatic (clinical observations) pigs. Sequence names NOT bold underlined are resistant pigs.
