# Supplementary material for: Resistance to African swine fever virus among African domestic pigs appears to be associated with a distinct polymorphic signature in the RelA gene and upregulation of RelA transcription
Source: Virol J. 2024 Apr 24;21:93. doi: 10.1186/s12985-024-02351-9 (PMC11041040; doi:10.1186/s12985-024-02351-9)
Supplement: Supplementary file 3 — Supplementary Material 3: Table S1: Prediction of disease-related amino acid substitutions by MutPred and PredictSNP sofwares [file 12985_2024_2351_MOESM3_ESM.docx]

**Table S1.** Prediction of disease-related amino acid substitutions by MutPred and PredictSNP sofwares.

|  | **MutPred prediction** | | | **Predict SNP** | |
| --- | --- | --- | --- | --- | --- |
| **SNPs** | **Actionable/Confident hypothesis** | **g-value** | **P-value** | **Confidence** | **Pathogenicity** |
| S374P | Gain of Intrinsic disorder | 0.701 | 0.0032 | 61 | Deleterious |
|  | Loss of Helix |  | 0.00052 |  |  |
|  | Gain of Loop |  | 0.04 |  |  |
|  | Gain of B-factor |  | 0.04 |  |  |
|  | Altered Transmembrane protein |  | 0.02 |  |  |
| S448T | Gain of Intrinsic disorder | 0.802 | 0.04 | 74 | Deleterious |
|  | Loss of Helix |  | 0.04 |  |  |
|  | Altered Transmembrane protein |  | 0.0032 |  |  |
|  | Altered Stability |  | 0.02 |  |  |
|  | Gain of N-linked glycosylation at N456 |  | 0.02 |  |  |
|  | Altered Transmembrane protein |  | 0.0085 |  |  |
|  | Loss of N-linked glycosylation at N456 |  | 0.02 |  |  |
| R462P | Loss of Loop | 0.690 | 0.01 | 65 | Deleterious |
|  | Gain of ADP-ribosylation at P460 |  | 0.01 |  |  |
|  | Altered Transmembrane protein |  | 0.02 |  |  |
|  | Gain of N-linked glycosylation at N456 |  | 0.02 |  |  |
| E495L | Gain of Intrinsic disorder | 0.761 | 0.0004 | 66 | Deleterious |
|  | Altered Disordered interface |  | 0.02 |  |  |
|  | Gain of Sulfation at Y496 |  | 0.04 |  |  |
| Q499P | Altered Disordered interface | 0.592 | 0.03 | 61 | Deleterious |
|  | Gain of Helix |  | 0.05 |  |  |
|  | Loss of Loop |  | 0.03 |  |  |
|  | Gain of Proteolytic cleavage at R502 |  | 0.0007 |  |  |

g-value: the probability for pathogenicity of amino acid substitutions. g-value > 0.50: pathogenic, g-value < 0.50: benign.

p-value:the probability for deleterious of amino acid substitutions. p-value: <-1 to 0: neutral; p-value: 0 to +1: deleterious

Amino acid substitutions highlited with g-value > 0.70 and p-value < 0.05; values are referred to as confident hypotheses.
