# Supplementary material for: Resistance to African swine fever virus among African domestic pigs appears to be associated with a distinct polymorphic signature in the RelA gene and upregulation of RelA transcription
Source: Virol J. 2024 Apr 24;21:93. doi: 10.1186/s12985-024-02351-9 (PMC11041040; doi:10.1186/s12985-024-02351-9)
Supplement: Supplementary file 4 — Supplementary Material 4: Table S2: Prediction of free energy change using MUPro andI-Mutant3 servers [file 12985_2024_2351_MOESM4_ESM.docx]

**Table S2.** Prediction of free energy change using MUPro and I-Mutant3 servers

| **Predicted damaging AA Substitution** | **Free energy (DDG) prediction** | | **I-Mutant 3.0(DDG Kcal/mole)** | |
| --- | --- | --- | --- | --- |
|  | **Scores** | **Mutation category** | **Score** | **Mutation category** |
| S9P | -1.314 | Decreased stability | -1.81 | Decreased stability |
| S84T | -1.992 | Decreased stability | -2.45 | Decreased stability |
| R97P | -0.779 | Decreased stability | -1.19 | Decreased stability |
| Q99V | -1.649 | Decreased stability | -2.04 | Decreased stability |
| H113Q | -0.898 | Decreased stability | -0.96 | Decreased stability |
| E130L | -1.493 | Decreased stability | -1.47 | Decreased stability |
| Q134P | -0.398 | Decreased stability | -1.03 | Decreased stability |

^a^ Free energy change ((DDG). If DDG value is < −0.5: Large Decrease of Stability; > 0.5: Large Increase of Stability.
